# Quality Improvement Study With Low-Cost Strategies to Reduce Neonatal Admission Hypothermia

**DOI:** 10.7759/cureus.40301

**Published:** 2023-06-12

**Authors:** PI Pragyan Pratik, Shilpa Krishnapura Lakshminarayana, Sahana Devadas, Anitha Kommalur, Sushma Veeranna Sajjan, Mallesh Kariyappa

**Affiliations:** 1 Department of Paediatrics, Vani Vilas Hospital, Bangalore Medical College and Research Institute, Bengaluru, IND

**Keywords:** skin-to-skin contact, kangaroo transport, quality improvement, neonate, hypothermia

## Abstract

Background

Admission hypothermia is still an underappreciated major challenge for new-born survival in low-resource settings. The WHO recommends skin-to-skin contact as the simplest and safest way for maintaining the body temperature even during transportation. Quality improvement initiatives for hospitalised new-borns have shown benefits like a reduction in neonatal morbidity and mortality. This study was undertaken in a resource-constrained public hospital in southern India with an aim to reduce neonatal hypothermia at admission to <20%.

Method

It was a prospective, quality improvement study undertaken over 20 weeks. All neonates born in the selected delivery room (DR), requiring transportation to the neonatal intensive care unit, were included. The primary outcome indicators were the mean axillary temperature of neonates measured upon arrival at the neonatal intensive care unit and the percentage of neonates with hypothermia at admission. Improving the thermoregulatory practices and ambient DR temperature to >25˚C, transportation by the kangaroo method, and a portable infant warmer (PIW) were implemented in three successive Plan-Do-Study-Act (PDSA) cycles.

Result

In the third PDSA cycle, the mean admission temperature (36.51˚C ±0.82) was significantly (p<0.0001) higher when compared with the baseline phase (35.41˚C ±1.09), and there was a significant (p<0.001) reduction in hypothermia (33.33%). The aim was achieved in the last two weeks of the third cycle with a reduction in hypothermia to 17.6%.

Conclusion

Implementation of appropriate thermoregulatory practices and low-cost strategies like the kangaroo method and PIW using quality improvement methodology significantly reduced admission hypothermia.

## Introduction

Neonatal hypothermia, defined as a drop in the body temperature below 36.5°C (97.7°F) [1], is ubiquitous in low- and middle-income countries (LMICs) with an overall prevalence ranging from 32% to 85% in hospital-based studies [2] and has been recognised as a significant cause of neonatal illness and death [1,3]. The WHO has published guidelines [1] on the warm chain, a set of 10 interlinked procedures to be followed at birth and in the initial few hours to prevent hypothermia in all new-borns. The recommended ideal delivery room (DR) temperature is 25-28°C. Neonatal transport is a significant source of cold exposure and a potential weak link in the warm chain [3,4]. Unlike improved neonatal care in developed countries [4,5], the lack of thermal protection and admission hypothermia is still an underappreciated major challenge for new-born survival in LMICs [2,4,6] where the main barriers are exorbitant price and the lack of reliable infrastructure or constant power source for adopting advanced warming technologies coupled with several high-risk practices in the health facilities [4-6]. Skin-to-skin contact (STSC)/kangaroo method effectively reduces the risk of hypothermia [1,3,7]. Kangaroo mother care (KMC) could be an alternative to conventional care in low birth weight infants, especially in resource-limited settings [8]. KMC during transportation is safe, effectively provides physiological and thermal stability [9-11], and has been recommended as a feasible and low-cost alternative to conventional transport [9,10]. The WHO [1,3] and the Government of India [12] emphasise continuous STSC and recommend KMC [1,3,13] as the simplest and safest way for maintaining body temperature during transportation. The 12th international conference on KMC [14] has also recommended KMC transport for stable neonates. Despite several benefits and recommendations, KMC transport has not been effectively implemented due to various barriers [14]. The use of economical and less sophisticated devices [5] could be helpful in the adoption and implementation of the WHO thermal care guidelines in low-resource settings. The WHO has developed the “Point-of-Care Quality Improvement” model, where the gaps in quality could be identified and scientifically addressed through root-cause analysis and the implementation of evidence-based practices through a team approach using the Plan-Do-Study-Act (PDSA) cycle [15]. PDSA cycles provide a structured learning approach used to test changes by planning, carrying out, and studying the results of a change before acting on them in the next cycle [15]. QI initiatives for hospitalised new-borns in LMICs have shown benefits like a reduction in neonatal mortality and morbidity [16]. This study was undertaken at a public hospital in southern India as significant admission hypothermia was observed during the monthly neonatal audits. The aim was to reduce hypothermia at admission to <20% from the baseline over 20 weeks using a QI initiative.

## Materials and methods

Setting

This study was undertaken at Vani Vilas Hospital, attached to Bangalore Medical College and Research Institute. It is one of the largest, tertiary-care public referral hospitals in southern India. The hospital has four DRs, four operating theatres, and a 50-bedded, level III neonatal intensive care unit (NICU) with two in-born units and one out-born unit. The hospital conducts around 17,000 deliveries and receives around 6000 NICU (about 80% are in-born) admissions annually. The time taken for transporting the neonate from the DRs to the NICU is around 10-15 minutes. Being a referral hospital with a high admission rate, both the human resource and infrastructure are constrained. The study was undertaken in one of the DRs, catering to about 6000 deliveries each year.

Design

This was a prospective study and a QI initiative undertaken over a period of 20 weeks (August 2019 to December 2019) after obtaining approval from the institutional ethics committee. The study consisted of baseline, implementation, and sustenance phases. The aim was to reduce hypothermia at admission from the baseline value to < 20% over 20 weeks. The baseline phase (Week 1-4) consisted of prospective data collection and identification of the key barriers in the appropriate thermoregulatory practices. This was followed by the implementation phase (Week 5-16) which included three (four weeks each) phases of intervention in the form of PDSA cycles. The sustenance was observed in the last four weeks (Week 17-20).

Sample

All neonates born in the selected DR during the study period, requiring transportation to the NICU (like gestational age < 34 weeks, respiratory distress, shock, asphyxia, bleeding, major congenital malformations, infant of diabetic mother, IUGR, etc.), irrespective of their gestational age or birth weight, were included after obtaining informed written consent from the family members. Neonates who died in the DR before transportation and cases with a refusal of consent were excluded. The gestational age and birth weight of neonates were recorded in a predesigned proforma and cross-verified with the computer records to ensure zero lacunae in the data collection.

Team

A QI team comprising a senior neonatologist, paediatrician, medical student, two paediatric residents, and three neonatal staff nurses was formed. The senior neonatologist took up administrative responsibilities. The paediatrician reviewed the literature and planned interventions. The medical student collected and analysed the data. The paediatric residents facilitated the new-born care at birth and transportation during the initial period of the study, the neonatal staff nurses measured the admission temperature, and later during the study, they additionally facilitated transportation also. The team held weekly meetings and reviewed the data. Each intervention was tested on a small sample, and the results were analysed and adopted if found to be effective. The outcome of the interventions was discussed among the team members every week, and modifications if required were incorporated. At the end of each cycle, the work of the team members was acknowledged, and champion members were rewarded. A continuous feedback loop was maintained.

Measures (process and outcome indicator)

The major interventions were the following: the first cycle focused on improving the existing thermoregulatory practices and maintaining the ambient DR temperature above 25˚C; the second cycle comprised the transportation of neonates utilising the kangaroo method; and in the third cycle, a portable infant warmer (PIW) was utilised for transportation. Ambient DR temperature, number of transports utilising the kangaroo method, and PIW were plotted on a graph and used as process indicators. The primary outcome indicators were the mean axillary temperature (MAT) in degree Celsius (˚C) of neonates measured upon arrival at the thermostable environment of the NICU and the percentage of hypothermia at admission. Throughout the study, the axillary temperature measured using a digital thermometer was recorded into a register by the staff nurse. Hypothermia was defined as a temperature < 36.5˚C and categorised into mild (36.0-36.5˚C) and moderate to severe hypothermia (< 36˚C) [1]. Hyperthermia was considered as a balancing measure to identify any inadvertent adverse effect from the interventions and defined as a temperature > 37.5˚C.

Baseline phase (Week 1-4)

**Figure 1:**
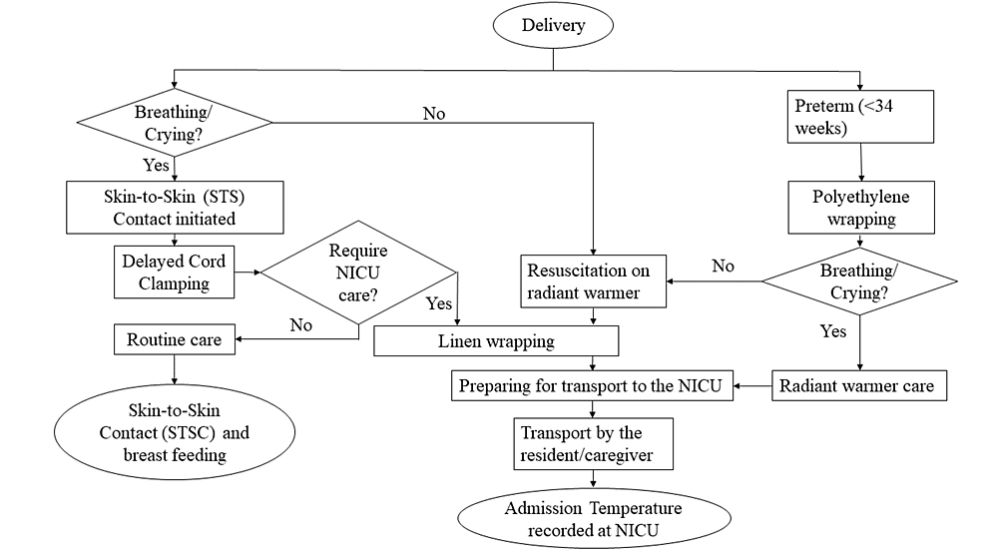
Process flow chart depicting the thermoregulatory processes in the DR and during the transportation

The team observed the existing thermoregulatory practices in the DR and during the transportation and constructed a process flow chart (Figure 1).

**Figure 2:**
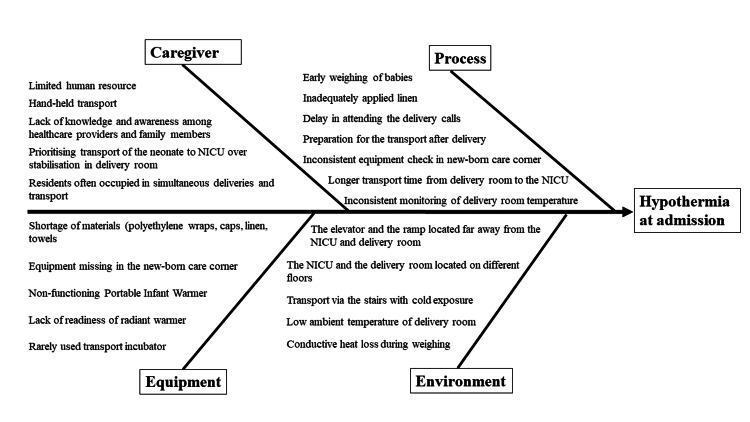
Root-cause analysis of hypothermia at admission using a fishbone diagram

Routine care was given over the mother’s abdomen, and resuscitation was done under the radiant warmer in the new-born care corner. Transportation was done by the hand-held method after wrapping the neonate with linen. A polyethylene cling wrap was used in neonates with < 34 weeks gestation. The root-cause analysis of admission hypothermia was done using a fishbone diagram (Figure 2) which helped in identifying the barriers in the thermoregulatory practices which potentially contributed to hypothermia. These were related to the facility setup, equipment, material and human resource availability, awareness, perception, and behaviour of the healthcare providers in the implementation of the warm chain.

**Figure 3:**
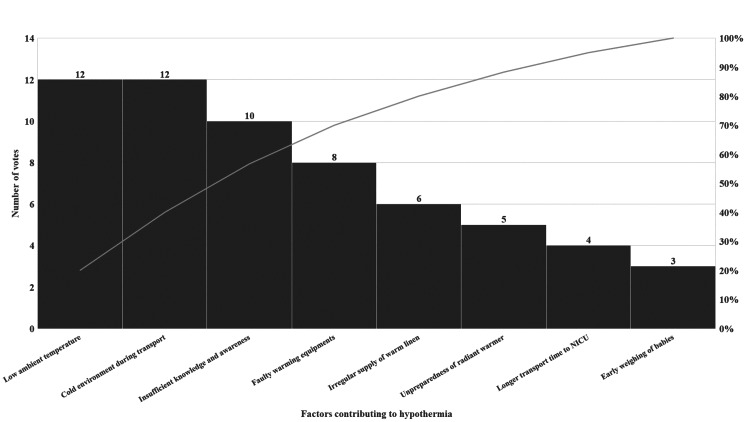
Pareto chart with the X-axis showing various factors potentially contributing to hypothermia and the Y-axis showing the frequency of these factors

The Pareto chart (Figure 3) revealed that 80% of the problem of hypothermia was predominantly due to 20% of the causes, namely low ambient DR temperature, heat loss during transportation, and the lack of knowledge and awareness among healthcare providers and family members.

Implementation phase

PDSA Cycle 1 (Week 5-8)

The team intervened to improve the thermoregulatory practices and to maintain the ambient DR temperature to > 25˚C. Information, education, and communication (IEC) materials on the warm chain components and the dangerous consequences of hypothermia were displayed in the waiting area, DR, and NICU. Focused group discussions involving staff nurses, residents, and doctors were organised and hands-on training on appropriate thermoregulatory practices using mannequins was imparted. The controller of the air-conditioner was adjusted, and it was emphasised on keeping the doors and windows closed so that the ambient DR temperature could be consistently maintained at > 25˚C. As the paediatric residents also had to attend deliveries in the other DRs, the team discussed with the hospital administrative authorities and subsequently made rearrangements in the neonatal staff nurses' rota and posted them in the DR for facilitating care at birth and transportation in addition to measuring the temperature of the neonate at the NICU. Consequently, the staff nurses took care of equipment in the new-born care corner, preheated the radiant warmer, documented the DR temperature, indented materials from the hospital store for consistent, adequate supply thus preventing stock-out periods, coordinated and facilitated transportation, and measured the admission temperature in the NICU.

PDSA Cycle 2 (Week 9-12)

**Figure 4:**
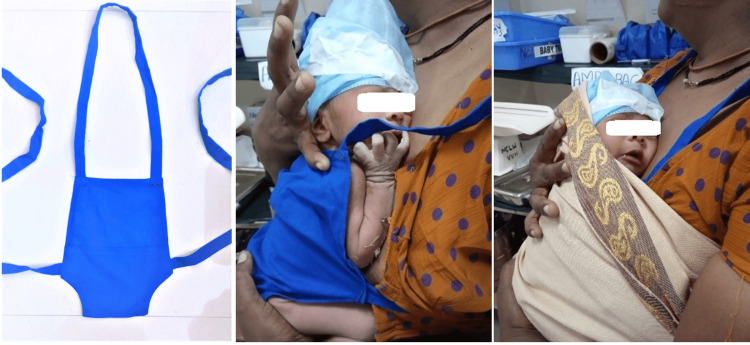
Kangaroo Mother Care bag and Kangaroo method of transport with cloth binder

The interventions of the first cycle were continued. Additionally, kangaroo transport (KT) was implemented for improving the thermal stability during transportation to the NICU. Transport of the neonate with STSC in the KMC position by the family member was planned. Due to safety concerns, it was decided to use KT for only haemodynamically stable neonates after explaining the transport procedure and obtaining informed written consent from the family member. Those who were unstable or refused consent for KT were transported hand-held. After discussing the logistics and ruling out the feasibility of utilising a gurney or a wheelchair for KT in the setup, the team planned KT by walking through the stairs via the nearest route to the NICU. Hands-on training about the technique of the KT was imparted to the staff nurses and residents using mannequins prior to commencing this intervention. The importance of hand hygiene was emphasised, and the technique of handwashing was demonstrated to the family member. In the initial transports, after handwashing by the family member and staff nurse, the neonate, wearing only a diaper and head cap, was placed by the nurse either over the family member’s bare chest or over the cloth worn by him/her after ensuring a minimal layer of clothing in between depending on his/her acceptability and privacy concerns and both were covered with a cloth. The staff nurse guided the family member to walk slowly and steadily toward the NICU. The nurse also assisted in taking the neonate out of the KMC position after arrival at the NICU and measured the axillary temperature. As the team and family members expressed apprehensions regarding the safety of the neonate, it was later decided to utilise the KMC bag which was being used for another QI project (Figure 4). Cost-effective, reusable cotton bags stitched by a local tailor were purchased. It was funded by a grant received from the Indian Academy of Paediatrics. The bags were used after autoclaving. KT utilising the KMC bags was implemented after providing hands-on training to the staff nurses and residents about the technique of its application. The neonate was placed upright inside the KMC bag which was tied around the chest and neck of the family member and secured in the KMC position with the help of a cloth binder (Figure 4) and prepared for transportation.

PDSA Cycle 3 (Week 13-16)

**Figure 5:**
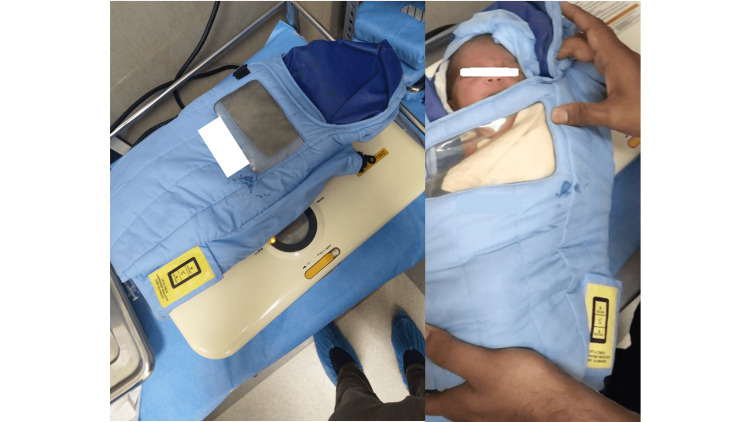
Portable infant warmer and neonate in the baby wrap

In addition to the interventions of the first and second cycles, a PIW was arranged by the hospital administrative authorities on request from the team members and utilised for transportation through Weeks 13-16. It is a small, lightweight, easy-to-carry, and clean sleeping bag-like model (Figure 5) which contained phase-change material that could be reheated either electrically or by pouring hot water into the compartment and could function without electricity for up to six hours. It was decided to use PIW for both stable and unstable neonates. The KT was utilised for stable neonates if the PIW was occupied for transport or if PIW had to be reheated. Hand-held transport was utilised when there was consent refusal for the KT and for unstable neonates if the PIW was preoccupied. The staff nurses and residents were trained hands-on about the technique of preparing the baby wrap before each transport, and the user manual was displayed in the DR. The baby wrap was sanitised using a disinfectant cotton swab and reused. The staff nurse facilitated the transport (Figure 5) and removed the neonate from the wrap after reaching the NICU and measured the temperature.

Sustenance phase

**Figure 6:**
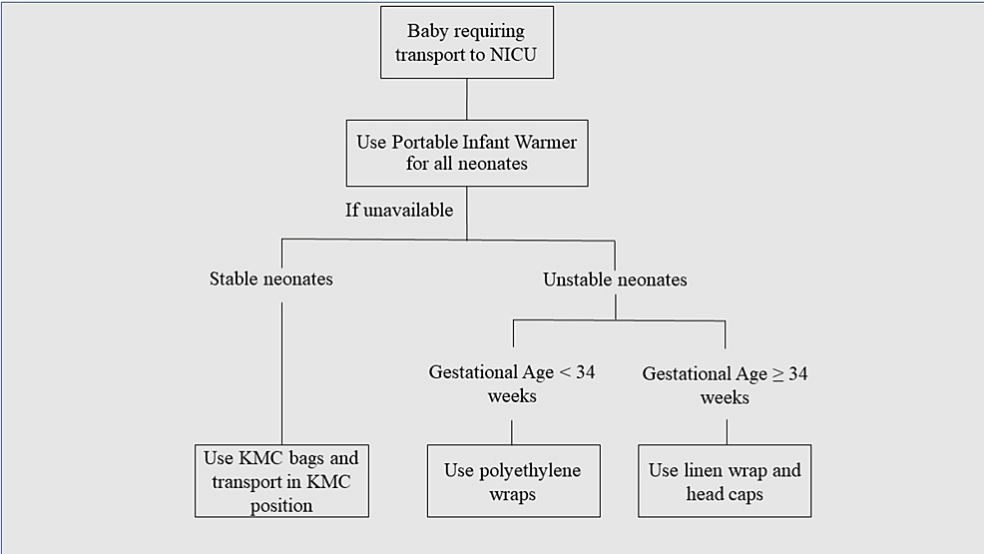
Protocol for neonatal transport inducted at the end of the study

Sustenance was studied over Weeks 17-20 and it was planned to analyse the effects further in monthly audits. A fixed protocol was constructed explaining the indication of various modes of neonatal transport (Figure 6).

Statistical analysis

The null hypothesis was that there would be no improvement in the MAT over time. The data was entered on an Excel sheet (Microsoft, Washington, USA) and analysed using SPSS Statistics version 29.0.0.0 (IBM Corp. Released 2022. IBM SPSS Statistics for Windows, Version 29.0. Armonk, NY: IBM Corp). The data was tested for normality by the Kolmogorov-Smirnov test. Time periods were categorised as baseline (four weeks before intervention), implementation (12 weeks of intervention), and sustenance phase (four weeks post-intervention) for comparison. All the quantitative data were expressed using descriptive statistics such as mean and standard deviation. The ANOVA test was conducted to compare the mean admission temperature between various groups, and the χ^2^ test of significance was used to test for changes between the pre- and post-implementation phases for categorical variables. A p-value of < 0.05 was considered statistically significant.

## Results

A total of 2,164 neonates were delivered during the study period. About 197 were eligible for transportation to the NICU of which 187 were included as 10 died in the DR (Table 1). The baseline characteristics were similar in all the phases (Table 1).

**Table 1:** Comparison of the baseline characteristics of the study population in the various phases of the study ^†^n= number of neonates ^‡^SD= standard deviation ^§^P-value calculated using ANOVA test

	Baseline phase	Implementation phase (PDSA cycle 1)	Implementation phase (PDSA cycle 2)	Implementation phase (PDSA cycle 3)	Sustenance phase	p-value^§^
Total live births (n^†^=2164)	246	456	515	520	427	
Neonates eligible for transportation to NICU (n=197)	30	48	39	41	39	
Neonates who died in the DR (n=10)	1	3	3	2	1	
Neonates with consent refusal (n=0)	0	0	0	0	0	
Total neonates included (n=187)	29	45	36	39	38	
Mean gestational age (SD^‡^)	33.4 (3.4)	34.6 (2.9)	34.9 (3.4)	35.7 (3.5)	35.4 (2.8)	0.054
Mean birth weight (SD^‡^)	2 (0.6)	1.92 (0.5)	2 (0.6)	2.1 (0.6)	2.1 (0.5)	0.430

**Figure 7:**
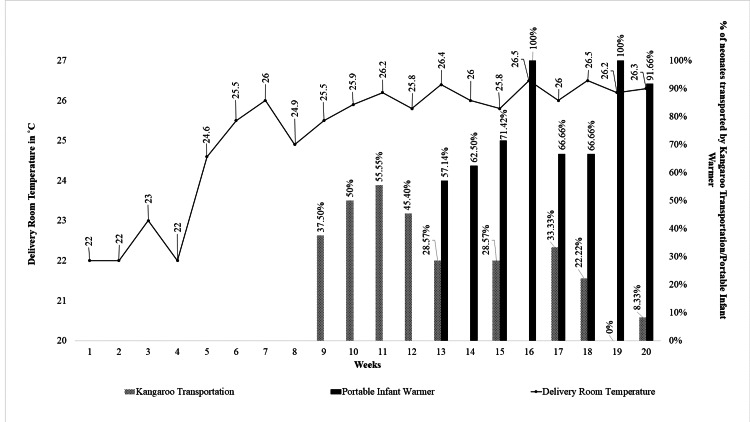
Process indicator depicting the extent of implementation of the interventions

The mean ambient temperature of the DR (Table 2, Figure 7) in the baseline phase was 22.25˚C. It increased to 25.25˚C (SD= 0.62), 25.85˚C (0.29), and 26.175˚C (0.33) in cycles 1, 2, and 3, respectively. In the sustenance phase, it was 26.25˚C (0.20).

**Table 2:** Mean ambient temperature of the delivery room and mean admission temperature, percentage of hypothermia, and moderate and severe hypothermia during the various phases of the study ^†^P-value calculated using ANOVA test ^‡^P-value calculated using chi-square test

	Baseline phase	Implementation phase (PDSA cycle 1)	Implementation phase (PDSA cycle 2)	Implementation phase (PDSA cycle 2)	Sustenance phase	p-value
Mean ambient temperature in ˚C (SD)	22.25 (0.5)	25.25 (0.62)	25.85 (0.29)	26.175 (0.33)	26.25 (0.20)	<0.0001^†^
Mean admission temperature in ˚C (SD)	35.41 (1.09)	36.04 (1.29)	36.47 (0.55)	36.51 (0.82)	36.55 (0.45)	<.0001^†^
% of hypothermia (<36.5˚C)	79.30%	55.55%	33.33%	33.33%	26.31%	<0.001^‡^
% of moderate and severe hypothermia (≤ 35.9˚C)	62.06%	22.22%	11.11%	15.38%	2.63%	<0.001^‡^

**Figure 8:**
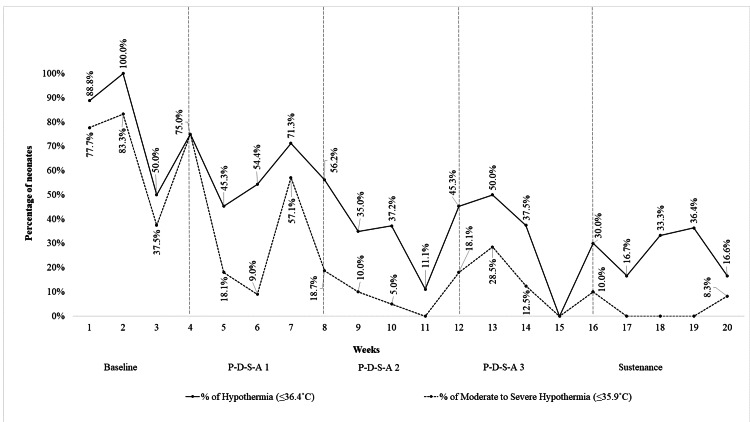
Line chart showing the percentage of neonates with hypothermia and moderate to severe hypothermia at admission

MAT, percentage of hypothermia, and moderate to severe hypothermia in the various study phases are as follows (Table 2, Figure 8).

In the baseline phase, 29 neonates were included of which 79.3% had hypothermia with 62% having moderate to severe hypothermia. The MAT was 35.41˚C (SD= 1.09).

In the implementation phase, 120 neonates were included of which 49 were included in cycle 1. In the second cycle, 36 neonates were included among whom 17 (47.22%) were transported using KT and 19 were transported hand-held (12 were unstable and 7 refused consent for KT). Thirty-nine neonates were included in the third cycle of which 28 (71.79%) were PIW transports, 6 (15.38%) were KT, and 5 were hand-held transports. The MAT was 36.04˚C (1.29), 36.47˚C (0.55), and 36.51˚C (0.82) in cycles 1, 2, and 3, respectively. The percentage of neonates with hypothermia was 55.55 and 33.33 in cycles 1 and 2, respectively, and in the third cycle, it remained at 33.33. The percentage of neonates with moderate to severe hypothermia was 22.22, 11.11, and 15.38 in cycles 1, 2, and 3, respectively. Reduction in hypothermia was observed across all the birth weight and gestational age categories with substantial reduction seen in the 1500-2500 grams category from the baseline 100% to 27.7% (p-value < 0.001) (Table 3). The MAT among neonates transported by KT (n=23) in the second and third cycles (36.63°C ±0.5) was significantly higher (p-value < 0.05) when compared to hand-held transports in the first cycle (36.04˚C ±1.29), and there was no significant difference (36.63°C ±0.5 vs 36.8°C ±0.5, p-value> 0.2) between those transported by KT and PIW.

**Table 3:** Percentage of hypothermia (<36.5 ˚C) at admission across all gestational age and birth weight categories ^†^n= number of neonates ^‡^P-value calculated using chi-square test

		Baseline phase (n^†^)	Implementation phase PDSA cycle 1 (n)	Implementation phase PDSA cycle 2 (n)	Implementation phase PDSA cycle 3 (n)	Sustenance phase (n)	p-value^‡^
Gestational age	<28 weeks	100% (1)	n=0	n=0	n=0	n=0	
	28-31 weeks	85.71% (7)	71.42% (7)	60% (5)	60% (5)	16.66% (6)	<0.001
	32-36 weeks	70.58% (17)	54.54% (22)	37.50% (16)	35.29% (17)	42.85% (14)	0.216
	37-41 Weeks	75% (4)	50% (16)	20% (15)	23.52% (17)	17% (18)	<0.001
Birth weight	<1000 gm	n=0	n=0	100% (1)	n=0	n=0	
	1000-1500 gm	44.44% (9)	78.50% (14)	40% (10)	57.10% (7)	33.33% (3)	<0.001
	1500-2500 gm	100% (13)	50% (24)	29.41% (17)	27.77% (18)	22.22% (27)	<0.001
	2500-4000 gm	71.42% (7)	28.57% (7)	25% (8)	28.57% (14)	37.5% (8)	<0.001

In the sustenance phase, the MAT was 36.55˚C (SD=0.45). About 26.31% of neonates were hypothermic with 2.63% having moderate to severe hypothermia.

A post-hoc analysis for pair-wise comparison was done, and it revealed that there was a statistically significant difference from the baseline to all the phases of implementation and sustenance phase and also among the different pairs compared (p-value < 0.05). No adverse events like falls, injuries, or hyperthermia were noted during the study.

In summary, the mean ambient DR temperature was significantly higher (p-value < 0.0001) in the third cycle (26.17˚C ±0.33) than in the baseline phase (22.25˚C ±0.5). The MAT of neonates was significantly higher (p-value= 0.00003) in the third cycle (36.51˚C, SD=0.82) than in the baseline phase (35.41˚C, SD= 1.09) (Table 2). There was a significant reduction (p-value < 0.001) in hypothermia (Table 2) from the baseline 79.3% to 33.33% in the third cycle. The aim of the study was achieved in the last two weeks of the third cycle with 17.6% of the neonates remaining hypothermic (Figure 8). There was a statistically significant (p-value < 0.001) reduction in moderate to severe hypothermia from the baseline 62% to 15.38% during the third cycle (Table 2). The result was sustained in the following month. Reduction in hypothermia was observed across all the birth weight and gestational age categories during the third cycle (Table 3). The MAT among neonates transported by KT was significantly higher (p-value< 0.05) when compared to hand-held transports, and there was no significant difference (p-value > 0.2) when compared to those transported in the PIW.

## Discussion

This QI study could achieve the aim of reducing admission hypothermia to < 20%, with a significant reduction across all the gestational age and birth weight groups and a reduction in moderate to severe hypothermia. This was one of the first QI initiatives undertaken at our centre. At a centre with 70% admission hypothermia (< 36˚C), a QI project showed improvement in the proportion of normothermic neonates from 27% to 75% and reduction in hypothermia from a median of 38% to 6% [17]. QI integrates hospital infrastructure with the process of providing safe, effective, and affordable patient care [15]. Considering the vast facility setup, high admission rate, and human resource shortage, QI was undertaken in only one of the DRs. In addition, it was feasible and safer to test the interventions in one DR. The DR, which catered to about 30% of the total deliveries, was selected as more neonates could be included. QI tools like the process flow chart, fishbone diagram, and Pareto chart helped in identifying the various factors contributing to hypothermia. As these were predominantly perception and behavioural issues coupled with ignorance, the initial interventions in the DR largely focused on participatory education and behaviour modification. Local rearrangements ensured sufficient availability of materials and human resources in the DR. The mean ambient DR temperature improved significantly. Once the appropriate thermoregulatory practices in the DR were established, the team intervened to improve thermal care during transport. The utilisation of the transport incubator was not considered feasible due to the higher admission rate and constraints in the human resource and facility infrastructure. Considering the various benefits, KT was implemented by involving the family members. This strategy also encouraged and reinforced family participatory care. Due to the infrastructural constraints, the family member providing KT had to stroll to the NICU. This also helped in reducing the transport duration by circumventing the waiting time for a wheelchair/gurney and also avoided the burden on the already constrained human resource. The reusable KMC bags secured the neonates during the KT. As it was a new intervention in the facility, KT was utilised only for stable neonates. The PIW was utilised for transporting both stable and unstable neonates. The PIW, a small, low-cost, lightweight model, with a baby wrap to carry the neonate, helped in an easy, fast, and safe transport. It could be easily cleaned and carried in hands without any additional aids. Furthermore, once fully heated, it could be used multiple times over the next few hours. Hence, it was very much feasible in a busy setting. Additionally, this model also served as a visual reminder and gave a behavioural message for promoting the STSC/KT. Overall, the DR interventions, simple and physiological KT with suitable modifications, and the PIW significantly reduced hypothermia in the setting. KT was as effective as the PIW and better than the hand-held transports in reducing hypothermia. The absence of adverse events reassured the safety of interventions. The results in the sustenance phase re-emphasized the effectiveness of the intervention strategies. The main challenges during this QI were while organising training sessions as the residents and staff nurses would often be busy and worked in shifts. Additionally, motivating and bringing change in the attitude and behaviour was not easy as this was one of the first QIs. Similarly, involving family members for the KT was challenging as they considered it awkward. Furthermore, anxiety and apprehensions regarding its safety had to be overcome. Regular weekly team meetings and discussions helped the team to find feasible strategies and overcome these challenges. The PDSA cycles helped in the effective implementation of the planned strategies.

To the best of our knowledge, this is the first QI study utilising the KT and a PIW and one of the first QI studies from a public institute in southern India. Low-cost intervention strategies using QI methodology and the results of this study are generalisable to other similar resource-constrained health facilities, as the majority of the contributing factors for hypothermia were similar to those observed in studies from Africa and South Asia [2]. Additionally, as QI methodology helps in finding solutions locally without major infrastructural changes, it could prove advantageous if adopted in similar settings to improve the quality of care. As new-born care has implications for their survival and future health [15], QI in their care is pivotal. This study could help policymakers in escalating the conduct of QIs at public healthcare institutions. It could also help convince and educate healthcare workers regarding the safety and efficiency of KMC transportation which in turn could expand the opportunity for family participatory care and also ease the burden on constrained human resources. The PIW could also be considered a low-cost, beneficial transport mode in similar settings. The strengths of this study were the utilisation of the KT and a PIW. Additionally, no additional human resources, infrastructure change, or major cost was incurred except for the procurement of the KMC bags and the PIW. Limitations were that, since a bundle-care approach using multiple strategies was collectively implemented in every cycle, the effect of a major contributory intervention in reducing hypothermia could not be analysed. The sustenance was studied for one month and could have been observed for a longer period. The morbidity, mortality profile, and final outcome of the neonate in the NICU with respect to admission temperature were not compared and were the scope of this study. Despite active measures, a small percentage of the neonates remained hypothermic, which should be analysed further in subsequent studies.

## Conclusions

The implementation of appropriate thermoregulatory practices and low-cost transportation strategies like the KT and PIW using QI methodology significantly reduced the admission of hypothermia and could be adopted by other resource-limited settings.
